# Development of 3D-Printing Filament from Recycled Low-Density Polyethylene (rLDPE) and High-Density Polyethylene (rHDPE) Composites Reinforced with Lignin Additive

**DOI:** 10.3390/polym18091028

**Published:** 2026-04-24

**Authors:** Nikolaos Pardalis, Sotirios Pemas, Nina Maria Ainali, Panagiotis A. Klonos, Apostolos Kyritsis, Konstantinos Spyrou, Dimitrios N. Bikiaris, Zoi Terzopoulou, Eleftheria Maria Pechlivani

**Affiliations:** 1 Laboratory of Polymer Chemistry and Technology, Department of Chemistry, Aristotle University of Thessaloniki, 54124 Thessaloniki, Greece; npardal@chem.auth.gr (N.P.); nsainali@chem.auth.gr (N.M.A.); pklonos@central.ntua.gr (P.A.K.); dbic@chem.auth.gr (D.N.B.); 2 Centre for Research and Technology Hellas, Information Technologies Institute, 6th km Charilaou-Thermi Road, 57001 Thessaloniki, Greece; sotpem15@gmail.com; 3 Dielectrics Research Group, Department of Physics, National Technical University of Athens, 15780 Athens, Greece; akyrits@central.ntua.gr; 4 Department of Materials Science and Engineering, University of Ioannina, 45110 Ioannina, Greece; k.spyrou@uoi.gr; 5 Laboratory of Industrial Chemistry, Department of Chemistry, University of Ioannina, 45110 Ioannina, Greece

**Keywords:** composites, lignin, bio-based fillers, recycled polyolefins, LDPE/HDPE blends, compatibilization, 3D-printing, fused filament fabrication (FFF)

## Abstract

This study investigates the development of sustainable composite materials using recycled low-density polyethylene (rLDPE) and high-density polyethylene (rHDPE) in an 80/20 mass ratio, incorporating kraft lignin as a bio-derived additive and polyethylene-graft-maleic anhydride (PE-g-MA) as a compatibilizer. Reactive melt mixing was employed to produce composites with varying lignin loadings (1, 3, 5, and 10 wt%). The structural, thermal, and mechanical properties and segmental dynamics of the materials were thoroughly examined using differential scanning calorimetry (DSC), infrared spectroscopy (IR), X-ray photoelectron spectroscopy (XPS), X-ray diffraction (XRD), thermogravimetric analysis (TGA), pyrolysis–gas chromatography/mass spectrometry (Py–GC/MS), tensile testing, scanning electron microscopy (SEM), and dielectric relaxation spectroscopy (DRS). The incorporation of lignin exhibited minimal disruption to the polymeric thermal transitions, while it boosted thermal stability, as confirmed by the TGA curves. According to the segmental dynamics findings, the glass transition temperature of the polymeric blend (−35 °C) was increased systematically with the addition of lignin by ~1–20 K. Tensile tests showed that the 1 wt% additive ratio demonstrated the optimal balance of strength and ductility. Morphological observations supported these findings, revealing uniform dispersion at low additive ratio and increased agglomeration at higher ratios. Based on its superior performance, the composite containing 1 wt% lignin was successfully extruded into filament suitable for 3D-printing. This study highlights the synergy of bio-based additives and recycled polymers in engineering high-performance materials, promoting circular economy principles and reduced environmental footprint through upcycling post-consumer waste into functional, valuable products.

## 1. Introduction

The accumulation of polyethylene plastic waste, especially derived from packaging, illustrates an urgent environmental and industrial issue. Polyethylene is the most widely used polymer worldwide, with annual global production exceeding 110 million tons, mainly divided into low-density polyethylene (LDPE) and high-density polyethylene (HDPE). These polyolefins are commonly employed in commodity and packaging applications, such as films, bags, bottles, containers, and household goods, owing to their flexibility, chemical resistance, low cost, and processability properties. However, their widespread post-consuming disposal contributes considerably to plastic pollution [1,2]. Mechanical recycling provides a cost-effective and environmentally sustainable way for revalorizing waste streams, but the collected plastic fractions typically contain mixtures of different polyolefins due to limitations in sorting technologies and contamination during waste management. As LDPE and HDPE are known to be thermodynamically immiscible, they are often found as mixed streams, causing poor adhesion and phase-separated morphologies that limit their mechanical properties [3]. This incompatibility is especially noticeable in blends containing high ratios of rLDPE and rHDPE, which display deteriorated strength and elongation compared to virgin materials [4].

**Figure 1:**
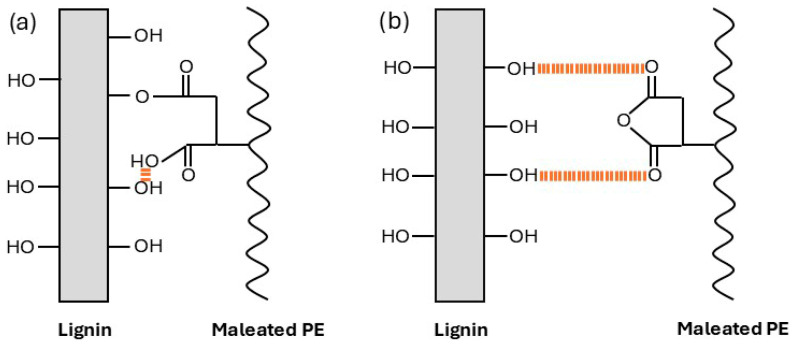
Possible interactions between lignin and maleated polyolefin via (**a**) esterification reaction and (**b**) hydrogen bonding.

To address the incompatibility of LDPE and HDPE in recycled blends, the incorporation of reinforcing additives and functional compatibilizers has emerged as a practical solution [5,6,7,8]. In this work, polyethylene-grafted maleic anhydride (PE-g-MA) is used as a compatibilizer due to its ability to enhance interfacial interactions [9,10]. The nonpolar polyethylene backbone of PE-g-MA enables good miscibility with both rLDPE and rHDPE, while its polar parts are able to interact with functional additives, containing polar groups [11]. Dikobe and Luyt [12] proposed the possible mechanism, mentioning both hydrogen bonding and esterification reactions between cellulose fibers and the maleated polyolefin (Figure 1). This functionality not only enhances compatibility between the polyolefin phases but also promotes the dispersion of polar additives in otherwise hydrophobic matrices. In this context, kraft lignin—a bio-based aromatic polymer with large surface area and abundance of hydroxyl groups—is introduced both as a green filler and a functional modifier, bridging the polar and nonpolar parts of the system [13,14].

Recent literature has shown promising effects of lignin-based composites on improving crystallinity, barrier properties, and thermal resistance in polymer matrices. For example, lignin has been employed in virgin polyethylene or polypropylene matrices to enhance mechanical properties and oxidative stability [15,16,17,18,19]. However, most prior work has focused on virgin polymer matrices or simple physical blends, with few reports addressing the integration of lignin into recycled, compatibilized polyolefin systems. Additionally, there is a lack of studies assessing how such bio-based reinforcements affect not only structure and thermal stability but also processing capability—a critical factor for advanced applications such as fused filament fabrication (FFF) [20,21]. Most previous research focuses on pellet-based mechanical analysis, without considering filament extrusion quality, dimensional control, or printability, which are essential parameters for upscaling into additive manufacturing workflows.

In this work, we aim to close the gap by evaluating the effect of kraft lignin and PE-g-MA compatibilizer on the structure and properties of recycled LDPE/HDPE blends. The composites were fabricated through melt mixing, with varying lignin contents (1–10% wt), while the compatibilizer concentration was kept constant. A full range of characterization techniques was employed to assess chemical interactions (IR), crystalline structure (XRD), thermal transitions and degradation (DSC, TGA, Py–GC/MS), dielectric spectroscopy (DRS), mechanical properties (tensile testing), and morphology (SEM). Special attention was given to pointing out the formulation with the most balanced performance across thermal, mechanical, and structural aspects.

Based on the comprehensive structure–property evaluation described above, the optimal formulation—identified as the one containing 1% wt lignin—was further selected to assess its processing capability. To this end, the material was successfully processed into a continuous filament using a single-screw extruder under controlled heating and cooling conditions. This step confirms the material’s viability as a feedstock for 3D-printing by transforming post-consumer polyolefin waste into valuable products. The work thus supports a circular materials strategy, leveraging both polymer waste and bio-derived additives to produce sustainable, functional composites.

## 2. Materials and Methods

### 2.1. Materials

Recycled low-density polyethylene (rLDPE) and recycled high-density polyethylene (rHDPE) were obtained in the form of pellets from DION ABETE (Thessaloniki, Greece). Post-consumer materials were milled into 10 mm flakes, washed in a flotation tank, dried, and finally placed in an extruder/pelletizer to create the recycled pellets. Kraft lignin (LIG) (BioPiva 100) was supplied by UPM (Helsinki, Finland). The hydrodynamic diameter, determined by dynamic light scattering (DLS), was 1589 nm, with a polydispersity index (PDI) of 0.26. For the compatibilization, polyethylene-grafted maleic anhydride (PE-g-MA) containing 0.5% wt maleic anhydride was purchased from Sigma-Aldrich (Darmstadt, Germany).

### 2.2. Preparation of Composites

Composites of rLDPE/rHDPE blends (80/20 *w*/*w*) with 1, 3, 5, and 10 wt% lignin and 5 wt% compatibilizer PE-g-MA [22], which was added to improve interfacial adhesion between the polymer matrix and lignin additive, were produced in a Haake–Buchler twin-screw co-rotating melt compounder. The 80/20 ratio was selected to achieve a balance between processability and mechanical performance. The LDPE-rich composition is expected to enhance melt flow and ductility, facilitating filament extrusion and 3D-printing, while the HDPE phase contributes to stiffness and mechanical strength. rLDPE and rHDPE pellets (diameter: 1 mm) were milled and pre-mixed with the LIG additive and 5% wt of the compatibilizer and dried at 80 °C overnight under vacuum. These materials were melt-mixed at 165 °C for 15 min, producing 4 batches of 10 g. A blank sample with no addition of LIG nor compatibilizer was also prepared for comparison purposes. All samples accompanied by the amounts of their components are presented in Table 1.

For further characterization, thin films were prepared by compression molding using a hot press at 180 °C. The obtained films had a thickness of approximately 0.5 ± 0.1 mm and were used for subsequent analyses.

### 2.3. Filament Fabrication and 3D-Printing

**Figure 2:**
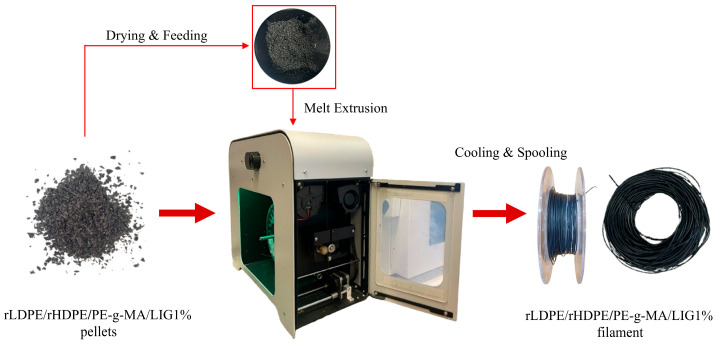
3devo Composer Filament Maker used for processing polymer blends into 1.75 mm filaments.

Two filaments were produced in total—one using the optimal mixture (rLDPE/rHDPE/PE-g-MA/LIG1%) and the other as reference filament using rLDPE/rHDPE/PE-g-MA. First, all materials were vacuum-dried overnight at 80 °C in pellet form. The dried materials were then fed into a precision desktop extruder 3devo Composer Series 350/450 (Utrecht, The Netherlands), which was used for filament fabrication. The machine includes four independent heating zones, a mixing screw extruder, an integrated cooling system for solidifying the extruded filament, and a rotating spool holder for collecting the final product. This process involves three primary stages: (i) gravity-fed intake of the pelletized mixture, (ii) thermal melting and mechanical mixing via a specialized screw to ensure LIG dispersion, and (iii) air-cooled extrusion followed by automated spooling. To ensure homogeneous melt flow and achieve a target filament diameter of 1.75 mm, the following temperature profile was applied: Heater 1: 170 °C, Heater 2: 160 °C, Heater 3: 150 °C, Heater 4: 145 °C. The polymer feed was progressively melted and conveyed using a screw speed of 6.3 rpm (Figure 2). The integrated cooling fans were set to 100% to ensure proper filament solidification. Under these conditions, the resulting filament exhibited diameter fluctuations within ±0.07 mm.

Based on previous studies and experimental work, a diameter range of 1.68 mm to 1.82 mm can be used for 3D-printing tensile specimens intended for mechanical testing to evaluate material properties. However, for printing larger or more complex objects, the lack of continuous diameter stability may pose limitations [20,23,24].

**Figure 3:**
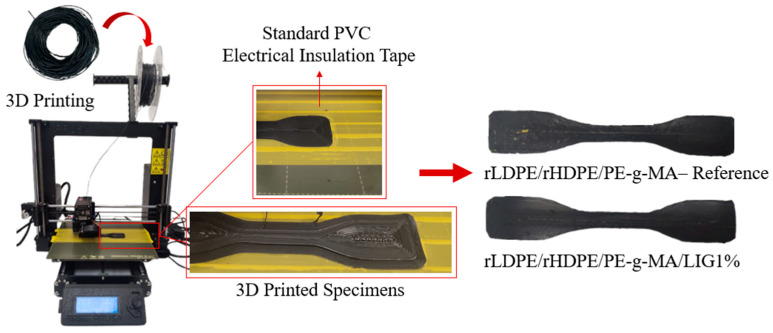
3D-printing process using the developed filaments.

The two developed filaments were both used in a commercial FDM 3D printer to demonstrate their printability and to evaluate their mechanical properties. To examine these properties, 10 specimens (5 for each filament type) were designed and printed according to the international standard ASTM D638, Type IV [25]. The specimens were designed using SOLIDWORKS^®^ CAD Software (2022 SP2.0 Professional version), and the exported STL files were processed through PrusaSlicer 2.5.2 to adjust the printing parameters and generate the G-code for the Original Prusa i3 MK3S+ FFF 3D printer (Prusa Research a.s., Prague, Czech Republic). A concentric infill pattern with 100% infill density was applied. All specimens were printed using the same printing parameters. The nozzle temperature was set to 260 °C, and the bed temperature to 60 °C. However, the exact bed temperature was not critical, as standard PVC electrical insulation tape was applied to the build plate to enhance bed adhesion. However, to overcome the poor natural adhesion of the rLDPE/rHDPE mixture to standard build surfaces, polyvinyl chloride (PVC) tape was applied to the build plate. While commercially designated for electrical insulation, the tape was utilized here strictly as a surface modifier. The chemical affinity between the PVC backing and the recycled polyolefin melt provided the necessary interfacial adhesion to prevent warping and part detachment, which otherwise rendered printing on conventional surfaces impossible. Figure 3 illustrates the 3D-printing process and the PVC tape application. The printing temperature of 260 °C was selected to ensure the optimal extrudability of the filament and to avoid clogging during printing. For the same reason, a 1 mm nozzle was used. This nozzle diameter was selected to mitigate backpressure and ensure consistent flow of the recycled polyolefin melt, which exhibits higher viscosity compared to virgin grades. Finally, the layer height was set to 0.3 mm.

### 2.4. Characterization Methods

#### 2.4.1. Infrared (IR) Spectroscopy

The FTIR spectra of all samples were obtained using a PerkinElmer FTIR spectrometer, model Spectrum One (Dresden, Germany). The IR spectra of these films were obtained in the absorbance mode and in the spectral region of 450–4000 cm^−1^ using a resolution of 32 co-added scans. The samples were in the form of thin films with thickness of approximately 150 µm and they were placed in holders with the appropriate dimensions of the spectrometer’s accessory.

#### 2.4.2. X-Ray Photoelectron Spectroscopy (XPS)

The X-ray photoelectron spectroscopy (XPS) measurements were obtained using a Kratos Analytical AXIS Ultra^DLD^ system (Kratos Analytical, Manchester, UK), featuring an aluminum monochromatic X-ray source (λ_Ka_ = 1486.6 eV), under ultra-high vacuum conditions (10^−9^ Torr). The survey spectra (0–1200 eV) were collected with an analyzer pass energy of 160 eV and 105 W applied to the X-ray gun. The high-resolution (HR) spectra were acquired with a pass energy of 20 eV set for the analyzer and 150 W applied to the X-ray gun, during a three-sweep scan. The XPS spectra were calibrated with reference to the C1s peak at 284.6 ± 0.2 eV from C–C bonds to correct for any surface charging effect. The background of the HR spectra was subtracted using Shirley or linear baselines, and the experimental curves were fitted using a combination of Gaussian (70%) and Lorentzian (30%) distributions. For quantitative analysis, the relative sensitivity factors (RSFs) of each element were obtained from the Vision 2.2.10 software database.

#### 2.4.3. X-Ray Diffraction (XRD)

XRD measurements were applied to study the crystalline structure of the composite materials in the form of a thin film. The XRD spectra were recorded at 25 °C, using the MiniFlex II XRD system (Rigaku Co., Ltd., Tokyo, Japan), with Cu Ka radiation (0.154 nm), over the 2θ range from 5° to 45°, and with a scanning rate of 1°/min.

#### 2.4.4. Differential Scanning Calorimetry (DSC)

Differential scanning calorimetry was performed in order to study the thermal transitions of the composites. The samples of 6 ± 0.1 mg sealed in aluminum pans were used in a PerkinElmer Pyris Diamond DSC differential scanning calorimeter (PerkinElmer, Solingen, Germany) calibrated with pure indium and zinc standards. The system included a PerkinElmer Intracooler 2 (PerkinElmer, Solingen, Germany) cooling accessory. Each sample was heated to 180 °C to erase previous thermal history and then cooled to room temperature at a rate of 20 °C/min under nitrogen atmosphere. Then, a second heating cycle of heating to 180 °C at the same scanning rate was performed to obtain the melting temperature (T_m_).

#### 2.4.5. Thermogravimetric Analysis (TGA)

Thermogravimetric analysis measurements were carried out using a NETZSCH STA 449F5 instrument (NETZSCH Group, Selb, Germany). The samples were placed in alumina crucibles and were heated under a 50 mL/min nitrogen flow and a heating rate of 20 °C/min, across a temperature range of 30–600 °C.

#### 2.4.6. Pyrolysis–Gas Chromatography/Mass Spectrometry (Py–GC/MS)

For Py–GC/MS analysis of the rLDPE/rHDPE composites, a very small amount of each material (≈1 mg) was “dropped” initially into the “Double-Shot” EGA/PY 3030D Pyrolyzer (Frontier Laboratories Ltd., Fukushima, Japan) using a CGS-1050Ex carrier gas selector. For the pyrolysis analysis (flash pyrolysis), each sample was placed into the sample cup, which was placed into the Pyrolyzer furnace afterward. The pre-selected pyrolysis temperature was set at 510 °C, based on the TGA data and the maximum degradation temperature, while the GC oven temperature was programmed at 50 °C for 2 min, followed by a stepped increase to 200 °C with a heating rate of 5 °C/min, where it was held for 8 min, and then the temperature was increased to 300 °C at a rate 20 °C/min, where it was held for 5 min. Sample vapors generated in the furnace were split (at a ratio of 1/50), a portion being moved to the column at a flow rate of 1 mL/min at pressure 53.6 kPa, and the remaining portion having exited the system via the vent. The pyrolyzates were separated using the temperature-programmed capillary column of a Shimadzu QP-2010 Ultra Plus (Kyoto, Japan) gas chromatogram and analyzed by the mass spectrometer MS-QP2010SE of Shimadzu (Kyoto, Japan). An Ultra-ALLOY^®^ metal capillary column from Frontier Laboratories LTD (Fukushima, Japan) was used containing a stationary phase of 5% diphenyl and 95% dimethylpolysiloxane, with a column length of 30 m and column ID of 0.25 mm. For the mass spectrometer, the following conditions were used: ion source heater of 200 °C, interface temperature of 300 °C, vacuum of 10^−4^–10^0^ Pa, *m*/*z* range of 40–500 amu (atomic mass unit), and scan speed of 10,000. The ion gas chromatogram and spectra retrieved through each experiment were subject to further interpretation through Shimadzu (NIST11.0) and Frontier (F-Search software 4.3) post-run software. Identification was based on the similarity percentage (minimum value of 80%) between the average mass spectra across the entire chromatogram.

#### 2.4.7. Dielectric Relaxation Spectroscopy (DRS)

The molecular dynamics of the polymers were assessed through DRS [26] in nitrogen atmosphere and in the temperature range between −140 and 50°C, by means of a Novocontrol BDS setup (Novocontrol GmbH, Montabaur, Germany). The samples were measured ‘as received’ and in the form of cylindrical disks of ~2 mm in thickness and 14 mm in diameter. To evaluate the dielectric response, the disks were placed between finely polished disk-like electrodes made of brass, forming a sandwich-like capacitor. The complex dielectric permittivity, ε* = *ε*′ − i × *ε*″, was recorded as a function of frequency in the range from 10^−1^ to 10^6^ Hz isothermally at various temperatures, with increasing steps of 5 or 10 K.

#### 2.4.8. Tensile Testing

The tensile properties of the prepared composites were measured using a Shimadzu EZ Test Tensile Tester, Model EZ-LX with a 2 kN load cell, in accordance with ASTM D882 [27], using a crosshead speed of 5 mm/min. In specific, dumbbell-shaped tensile test specimens (central portions 5 × 0.5 mm thick, 22 mm gauge length) were cut using a Wallace cutting press from samples in film form. At least five measurements were taken for each sample, and the results were averaged to obtain the mean values of Young’s modulus, tensile strength at break point, and % elongation at break point.

In addition, tensile properties of the 3D-printed samples were further evaluated. Dumbbell-shaped specimens were produced in accordance with the ASTM D638, Type IV geometry (central gauge length of 25 mm, thickness ~4 mm). The tests were carried out on the same Shimadzu EZ Test Tensile Tester, employing a crosshead speed of 50 mm/min, as recommended for this specimen type.

#### 2.4.9. Scanning Electron Microscopy (SEM)

The morphology of cryo-fractured surface of the composites was observed using a JEOL JMS 7610F (JEOL, Freising, Germany) scanning microscope. The operation was carried out at 5 kV. Before placing the samples in the vacuum chamber, they were sputtered with carbon to ensure conductivity.

## 3. Characterization and Discussion

### 3.1. Composite Characterization

#### 3.1.1. Structural Characterization

**Figure 4:**
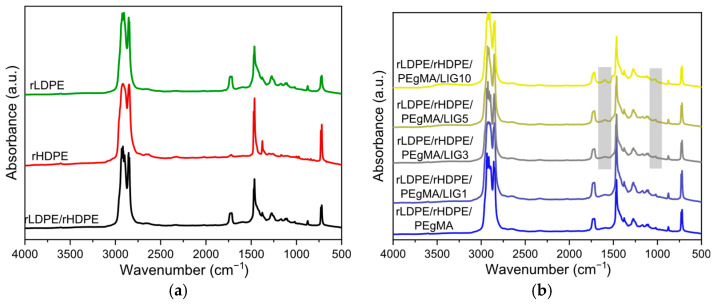
IR spectra of (**a**) rLDPE (green); rHDPE (red); their 80/20 blend (black); and (**b**) composites with varying lignin content.

The IR spectra of the composites in the form of pressed films (thickness: 0.5 ± 0.05) were examined in order to identify the chemical structure and possible interactions in the composites. Both neat rLDPE and rHDPE spectra (Figure 4a) show the typical polyethylene bands at 2914, 2848 cm^−1^, caused by the C–H stretch, and at 1468 cm^−1^, attributed to the C–H bend. A 720 cm^−1^ peak is also usually found in PE samples due to long, uninterrupted –CH_2_– sequences. For rLDPE, peaks observed at 1734 cm^−1^ and 1270–870 cm^−1^ are assigned to C=O and C–O–C vibrations respectively, which indicate the possible oxidation that occurred during thermal recycling [28,29]. The IR spectrum of the blend reveals the absorption bands of both components, indicating successful blending.

Similarly, the compatibilized sample also shows the relevant peaks of both polymers, confirming effective integration (Figure 4b). In composites with 1% and 3% wt LIG, the characteristic lignin bands are not clearly distinguishable, possibly due to the low concentration. On the other hand, at 5% and 10% wt LIG loadings, some filler-related features become more visible. They include the broad hydroxyl –OH stretch at ~3400 cm^−1^, as well as peaks around 1600 cm^−1^ and 1512 cm^−1^, corresponding to aromatic skeletal vibrations, and at 1030 cm^−1^, associated with the C–O stretch of alcohol and ether groups. This implies an increasing effect of filler in the composites’ structure as its ratio increases [30]. However, there is no clear evidence of interactions of PE-g-MA with lignin, as no peak shifts or new peaks are observed. This is due to the low amount of compatibilizer or the fact that these reaction footprints are hidden by the oxidation of the recycled polymers.

For this reason, X-ray photoelectron spectroscopy (XPS) was employed to identify the surface functional groups and elucidate the possible interactions between lignin and maleated polyolefin. Two potential interaction mechanisms were considered, namely (i) esterification reactions and (ii) hydrogen bonding. The analysis was conducted on the rLDPE/rHDPE/PE-g-MA/LIG1%, rLDPE/rHDPE/PE-g-MA/LIG5%, and rLDPE/rHDPE/PE-g-MA/LIG10% samples. Deconvolution of the high-resolution C1s spectrum of rLDPE/rHDPE/PE-g-MA/LIG1% reveals four distinct components. The main peak at 284.6 eV is attributed to C–C/C–H bonds associated with the polymer backbone of LDPE/HDPE, as well as contributions from the aromatic structure of lignin and PE-g-MA, accounting for 78.7% of the total carbon content. A second component at 285.1 eV is assigned to C–OH groups originating from lignin, representing 14.9% of the spectrum. The third peak, centered at 286.0 eV, corresponds to C–O–C functionalities, indicative of ether linkages present in both lignin and PE-g-MA, thereby confirming their incorporation within the polymer matrix. Finally, a fourth peak at 288.3 eV is attributed to C=O groups associated with the maleic anhydride moieties of PE-g-MA. Similar functional groups are observed in the high-resolution C1s spectra of rLDPE/rHDPE/PE-g-MA/LIG5% and rLDPE/rHDPE/PE-g-MA/LIG10% (Figure S1b,c). The primary difference among the samples lies in the relative contribution of hydroxyl (C–OH) groups. Notably, the rLDPE/rHDPE/PE-g-MA/LIG5% sample exhibits the highest C–OH content (17%), suggesting a higher degree of interfacial interaction or grafting efficiency at this composition. However, the differences in the percentage of C–OH bonds for all samples are very low, as presented on Figure S1a–c. Importantly, no additional peaks corresponding to ester linkages are observed, indicating that esterification does not occur to a significant extent under the applied conditions. Instead, the interactions between lignin and PE-g-MA are predominantly governed by hydrogen bonding, as supported by the presence and variation of oxygen-containing functional groups in the C1s spectra.

The O1s photoelectron spectra (Figure S2) are consistent with the C1s analysis, confirming the presence of C–O, C–O–C, and C=O functional groups in the final materials. In addition, a minor component observed at higher binding energies (~534 eV) for all samples can be attributed to adsorbed water, which is commonly associated with the hydrophilic nature of the materials.

**Figure 5:**
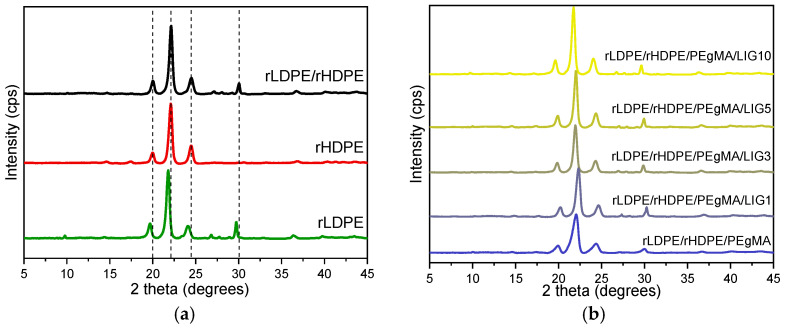
XRD patterns of (**a**) rLDPE (green), rHDPE (red), and their 80/20 blend (black), (**b**) composites with increasing lignin content.

XRD measurements were performed on the prepared films at room temperature to evaluate the crystalline structure of the rLDPE/rHDPE blends and their composites. XRD patterns of rLDPE, rHDPE, (Figure 5a), and their blend exhibited characteristic reflections at 2θ ≈ 21.5° and 23.8°, corresponding to the (110) and (200) planes of the orthorhombic polyethylene lattice. A third peak close to 20° is also present, attributed to disordered crystalline regions. These features are consistent with semicrystalline PE structures. As for rLDPE, it presented additional peaks at ~28°, ~29°, and 29.5–30°, which are not typically associated with pure polyethylene. These features may originate from additive residues, contaminants, or oxidative degradation products, supporting IR graphs [31,32]. The XRD patterns of the composites (Figure 5b) display the same characteristic diffraction peaks without noticeable shifts or significant intensity variations, supporting that the incorporation of lignin did not alter the crystalline structure of the matrix.

#### 3.1.2. Thermal Behavior and Stability

**Figure 6:**
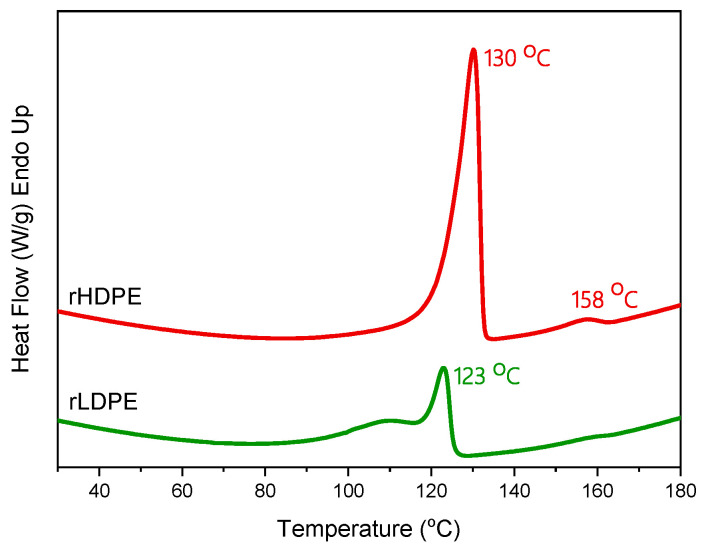
DSC thermograph of rLDPE (green) and rHDPE (red).

DSC measurements were taken to study the thermal properties of the composites. The recycled LDPE (Figure 6) revealed a main melting peak within the expected range, approximately at 123 °C [17], during the second heating cycle. However, the curve also displays a broad endothermic event spanning from 100 to 120 °C that suggests structural heterogeneities, a common phenomenon in recycled polymers. One possible explanation is the presence of other polymers or impurities, such as small amounts of EVA, which could influence the melting behavior. Additionally, the sample likely exhibits a broad molecular weight distribution, leading to a melting process that occurs over a wider temperature range. The repeated processing during recycling may have also induced thermal degradation, causing chain scission or oxidation, which in turn affects the material’s molecular weight and results in a less defined melting peak.

As for the recycled HDPE, DSC shows a strong melting peak at 130 °C, which corresponds to the melting of crystalline regions of HDPE, and a weak one at 158 °C. This small secondary peak strongly indicates the presence of PP contamination within the recycled stream. Such contamination is very common in recycled polymers and may affect both the thermal behavior and the mechanical performance of the composites [33].

**Figure 7:**
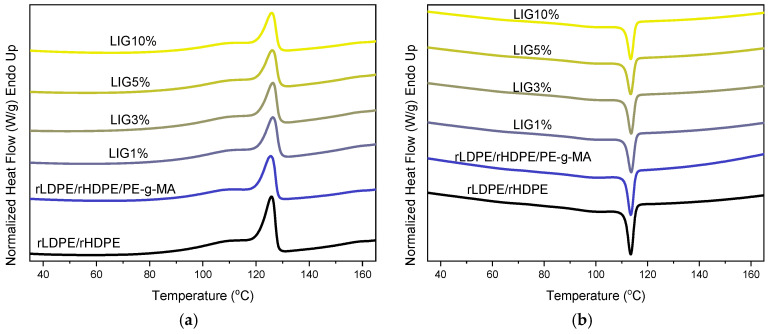
DSC thermographs of (**a**) composites’ 2nd heating and (**b**) composites’ cooling.

As for the composite samples, DSC graphs of the second heating and the cooling processes are exhibited in Figure 7. The melting and crystallization behavior of the materials were analyzed in the context of their individual components (rLDPE and rHDPE). Since rLDPE has a melting peak at 123 °C and rHDPE has a broad melting peak at 130 °C, the melting peak at 125.7 °C for their blends was expected. This peak remains unchanged in the rLDPE/rHDPE/PE-g-MA sample (125.6 °C) and is shifted to a slightly higher temperature (126 °C) in all composites without any effect of lignin amount. Similar observations were also recorded the for the crystallization temperature (Tc) that was recorded during cooling of the sample melts. In all composites, a Tc was recorded at about 114 °C, which is identical to that of the compatibilized rLDPE/rHDPE/PE-g-MA sample, without variations.

The fact that the blank sample (rLDPE/rHDPE) showed similar thermal transitions to the composites containing LIG (Table 2) suggests that the additive does not significantly influence the crystalline structure and thermal characteristics of the blends. Instead, the observed thermal transitions are primarily dictated by the interactions between the two polymers, with the melting and crystallization temperatures representing a combined effect of the two phases rather than a nucleating effect from the additive [13,14].

Table 3 shows the thermal characteristics derived via DSC. The uncompatibilized rLDPE/rHDPE blend showed a ΔHm of 43 J/g, intermediate between rHDPE (104 J/g) and rLDPE (38 J/g), as expected for polymer blends where each phase crystallizes independently [31,32]. The addition of 5% wt PE-g-MA compatibilizer resulted in a moderate increase in ΔHm (66 J/g) (and corresponding ΔHm), implying improved crystallizability, possibly due to increased interfacial adhesion and more efficient chain packing between the polymer phases [34,35]. In addition, the compatibilizer itself has lower molecular weight compared to rHDPE and rLDPE, and this could enhance the crystallization of the blend. Regarding the composites, the increase in ΔHm was not as prominent as in the rLDPE/rHDPE/PE-g-MA sample, implying that the presence of lignin might have inhibited crystallization due to restricted mobility.

**Figure 8:**
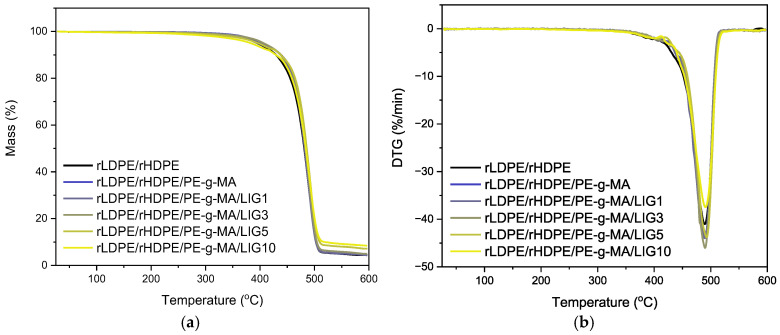
(**a**) Mass loss (%) and (**b**) DTG for all samples across the temperature range of 30–600 °C under nitrogen atmosphere.

Thermogravimetric analysis (TGA) was conducted to evaluate the thermal stability and decomposition behavior of the neat blend and its composites. Figure 8 shows that all rLDPE/rHDPE composites have good thermal stability, with onset degradation temperatures (T_onset_, determined by the intersection of the baseline and the tangent to the TGA curve at the point of maximum degradation rate) starting around 460–466 °C and maximum degradation rates (T_DTG_) occurring at around 489–492 °C [16,28]. Interestingly, while the thermal degradation values remain basically constant, a progressive increase in residual mass is observed with increasing LIG ratio (from 4.4% for the neat rLDPE/rHDPE to 8.4% at 10% wt LIG). This trend can be attributed to the higher char-forming tendency of lignin due to its aromatic and oxygenated structure, which is thermally more stable and resistant to volatilization. It is well known that flame retardancy is connected to the percentage of char formation, as the reduces the combustion rate of polymeric materials because it can delay the oxygen to reach the combustion zone [17,36].

To explore the degradation process for the prepared composites based on the rLDPE/rHDPE matrix, analytical pyrolysis was further employed. Analytical instrumentations based on pyrolysis have been highlighted as effective techniques for clarifying the degradation pathways of several polymers and their composites, as valuable data on these complex polymeric matrices can be derived from the examination of the pyrolysis products at a molecular level [37]. In detail, each group of macromolecules produces characteristic compounds after selective bond cleavage reactions induced by the thermal energy of pyrolysis. Once these small volatile compounds are formed, they are separated by GC and then detected by MS, providing structural information on the sample’s chemical composition. Herein, Py–GC/MS analysis was applied to evaluate whether the incorporation of LIG into the recycled rLDPE/rHDPE matrix affects the distribution of its volatile thermal degradation products.

**Figure 9:**
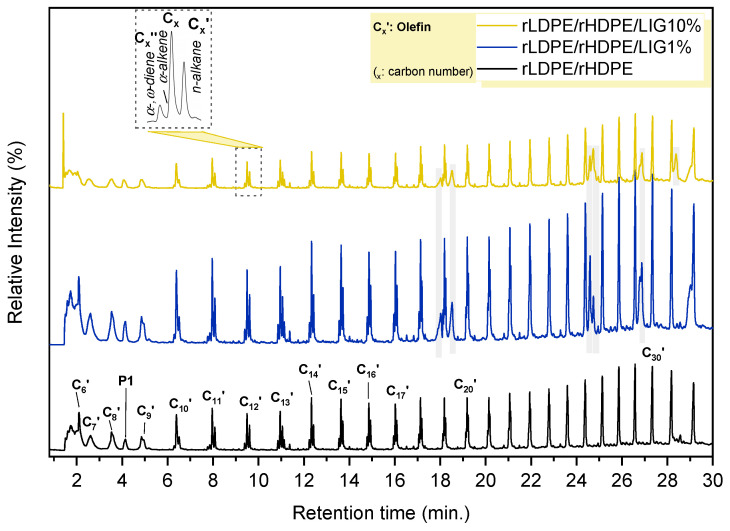
Total ion chromatograms (TICs) of the neat rLDPE/rHDPE blend and its composite containing 1 wt% and 10 wt% LIG after flash pyrolysis.

The total ion chromatograms (TICs) of the studied samples are depicted in Figure 9. All the TICs exhibited a series of repeating hydrocarbon peaks typical of PE cracking, which is characterized by a dense homologous distribution of hydrocarbon triplet peaks. The above profile is in agreement with the well-established thermal degradation mechanism of PE polyolefines, where the principal process includes random chain scission on C–C bonds along the polymer backbone, thus forming hydrocarbon radicals further undergoing β-scission and hydrogen transfer reactions. These reactions mainly lead to the formation of alkanes (C_x_, where x is the carbon number), alkenes (C_x_′), and α,ω-dienes (C_x_″) across a broad C-number range [38,39,40], extending from approximately C5 to C32 for our case. Representative triplets such as 1,19-eicosadiene (R_t_ = 19.1 min), 1-eicosene (R_t_ = 19.2 min), eicosane (R_t_ = 19.3 min) and 1,20-heneicosadiene (R_t_ = 20.0 min), 1-heneicosene (R_t_ = 20.1 min), heneicosane (R_t_ = 20.3 min) confirm the dominance of polyethylene-type radical cracking reactions. In addition to the hydrocarbon products, different oxygen-containing products were also detected, predominantly in the late retention times [41]. The above compounds contained long-chain alcohols, such as octacosanol (R_t_ = 26.8 min), 1-hentetracontanol (R_t_ = 24.6 min), and 1,30-triacontanediol (R_t_ = 28.4 min). These products may be attributed to the presence of minor non-polyolefinic components in the recycled feedstock, including, for instance, additives and wax-based processing aids, as well as oxidation products produced throughout the service life and recycling processes [42]. It is noteworthy to mention that, despite the fact that major volatile compounds originate from the polyethylene degradation, a characteristic signal at R_t_ = 4.1 min (P1), as well as other peaks (detected at R_t_ = 11.4 min for P2 and R_t_ = 18.5 min for P3) attributed to branched hydrocarbons, were also identified and are consistent with the presence of PP impurities in the recycled feedstock; a fact that is in well-accordance with the DSC results [43].

Owing to the fact that the TICs for all the studied samples are dominated by olefinic cracked products and there are no distinct LIG-based marker compounds that could be confidently identified through the library matching, the discussion on the results was based on a semi-quantitative evaluation of the integrated peaks. The latter behavior is plausible when studying polymer/lignocellulosic blends, where the simultaneous and mixed pyrolysis occasionally results in chromatograms dominated by the hydrocarbon derived by the polymers’ degradation rather than the emergence of new compounds [38]. In order to facilitate the comparison between complex chromatograms, the TICs were divided into three characteristic elution regions, including an early volatile region (R_t_ = 0–5 min), an intermediate region (R_t_ = 5–19 min), and a late region (R_t_ = 19–30 min), with the corresponding intensity fractions having been calculated relative to the total signal within the 0–30 min analysis frame. The resulting intensity fractions (f) were calculated based on the following Equation (1):

(1) $$f_{early}=\frac{A_{0–5}}{A_{0–30}},\;\;f_{mid}=\frac{A_{5–19}}{A_{0–30}},\;\;f_{late}=\frac{A_{19–30}}{A_{0–30}}$$

As for the neat rLDPE/rHDPE blend, it presented a fractional composition profile concentrated mainly in the late regions, with f_late_ = 0.53, whereas the early stage of decomposition accounted for f_early_ = 0.17 and the intermediate region for f_mid_ = 0.29. This dominance of the late retention time fraction is in compliance with the well-established thermal decomposition of PE, generating a homologous distribution of hydrocarbons encompassing long-chain alkenes/alkanes and higher boiling point oligomeric fragments, which typically elute in the later part of the TIC. After the incorporation of lignin at the polyolefinic backbone, systematic changes in the total elution profile of the samples were detected. Specifically, the composite containing 1 wt% LIG (rLDPE/rHDPE/LIG1%) presented a minor decrease in the early R_t_ fraction (f_early_ = 0.14) and a moderate reduction in the late fraction (f_late_ = 0.49) when compared with the neat blend, whereas the f_mid_ values remained comparable (f_mid_ = 0.28). The most noticeable differences were displayed for the 10 wt% LIG composite (rLDPE/rHDPE/LIG10%), in which the early fraction increased to f_early_ = 0.2 and the late fraction decreased substantially to f_late_ = 0.42, along with a reduction at the intermediate window to f_mid_ = 0.24. All the information presented above implies that the increasing lignin loading within the composites progressively shifts the detected TIC signal toward earlier R_t_, as well as to a relative suppression of the late-eluting fraction.

Interestingly, the decrease in the late-eluting region, which was observed with increasing lignin content, could be directly linked with the char-forming character of lignin and the higher residual mass (%), as also observed in the TGA results. According to the available literature, lignin undergoes thermally induced condensation and cross-linking reactions, thus favoring the formation of a solid carbonaceous residue rather than volatile compounds [44,45]. In the light of the information presented above, the insertion of lignin within the polyolefin matrix is expected to redirect the thermal degradation pathway toward a condensed phase, facilitating the increase in solid residue, and, thus, to lower relative contribution of high-boiling volatile molecules that would, under different conditions, elute within the late region of the chromatogram [45,46,47]. It is noteworthy to highlight that the absence of distinct lignin characteristic peaks in the chromatogram does not contradict the incorporation of lignin within the polymeric matrix, but it implies the presence of three possible scenarios, as follows: In the selected pyrolysis temperature, fragments derived from lignin either remain below the detection threshold of our methodology due to the predominant and abundant presence of polyolefin pyrolysates, they co-elute with the plenty hydrocarbon peaks, or they are converted into non-volatile condensed compounds in a preferential mean. Consequently, the analytical pyrolysis results along with the TGA residual mass results suggest that the incorporation of lignin principally affects the mode of thermal degradation by improving char formation, rather than importantly altering the hydrocarbon cracking profile of the polyolefin matrix, as also shown in previous studies [47,48].

#### 3.1.3. Dielectric Relaxation Spectroscopy (DRS)

**Figure 10:**
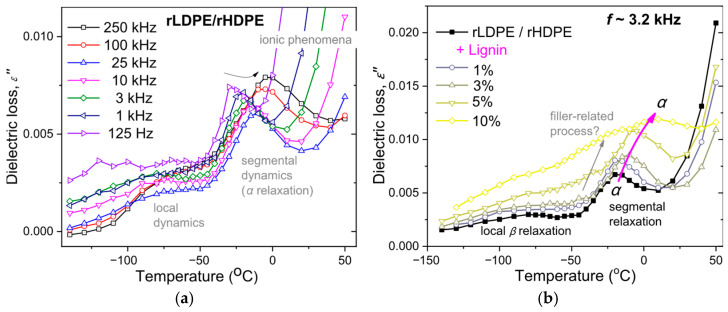
(**a**) The temperature dependence of ε″ across various frequencies (isochronal plots) in neat rLDPE/rHDPE. (**b**) The ε″(Τ) dependences shown comparatively for all samples at the selected frequency of ~3.2 Hz.

The sophisticated technique of DRS was employed to assess molecular mobility, particularly to assess segmental dynamics. The latter is related to the glass transition, which cannot be clearly recorded by calorimetry in highly crystalline polymer such as LDPE [49]. In DRS, the imaginary part of dielectric permittivity, *ε*″ (dielectric loss) is recorded and it is shown here as a function of temperature for various frequencies in Figure 10. Therein, the relaxation modes originating from molecular mobility are recorded as peaks. At the lower temperature side of Figure 10a, the local relaxation of PE is recorded, usually referred to as *β* relaxation.

At higher temperatures, namely, above −50°C, the dielectric signal increases significantly and the strong peak of *α* relaxation is recorded. *α* relaxation is the dielectric analog of the glass transition and it is believed to screen the relaxation of dipole moments located perpendicularly to the polymer chain backbone [26]. For further increased temperatures, *ε*″ increases sharply. The effect originates from the mobilization of ions; moreover, various ionic phenomena (ions transport through the polymer matrix, interfacial charge entrapments, etc.).

**Figure 11:**
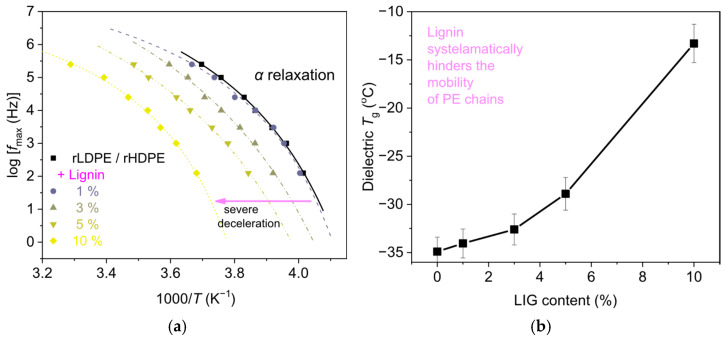
(**a**) The reciprocal temperature dependence of the frequency maxima, fmax, of α relaxation for all samples. The lines connecting the experimental points are fittings of the Vogel–Tammann–Fulcher–Hesse equation. (**b**) The lignin content dependence of the dielectric Tg as estimated by the time scale of α relaxation.

For fixed frequency of the AC field, it is observed in the comparative traces of Figure 10b that the addition of lignin induces a systematic increase in the overall dielectric signal, along with an individual dipolar contribution (peak addressed as ‘filler-related’). The effect is a clear although indirect indication of good dispersion of lignin within the polymeric matrix. The most significant result here is the recording of *α* relaxation of PE. In Figure 10b, the addition of lignin leads to a monotonic migration of the *α* relaxation peak toward higher temperatures. This is clearly an effect of ‘hindering/deceleration’ of the polymer chain mobility. The systematic effect is an additional indication for the good lignin dispersion. Employing a widely known method of analysis of the dielectric data [50,51,52] the time scale of *α* relaxation was evaluated and is depicted here in Figure 11a. Therein, the curved time scale of *α* relaxation is denoting of its ‘cooperative character’, manifesting that the relaxation is indeed related to the glass transition. Then, said deceleration of *α* relaxation in the presence of lignin is clear for the full range frequency–temperature range of recording.

Finally, by extrapolating the data of *α* relaxation to the equivalent frequency of conventional calorimetry (*f*_eq,DSC_ ~ 10^−2.8^ Hz, due to the relaxation time at *T* = *T*_g_ equalling 100 s), the ‘dielectric glass transition temperature’, *T*_g,diel_, was estimated. The results are presented as a function of lignin loading in Figure 11b. In the neat rLDPE/rHDPE blend, *T*_g,diel_ equals −35 °C. Interestingly, for 1, 3, 5, and 10% LIG, *T*_g,diel_ increases by 1, 2, 5, and 21 K, respectively. Despite any uncertainty involved, the increase is significant and systematic, denoting the hindering of the ‘amorphous’ polymer chains mobility at the addition of lignin. This suggests that a significant number of lignin entities ‘interact’ (bonding) with amorphous PE chains (e.g., of HDPE), or/and the effect is an indirect effect of crystallinity. The latter can be understood in the sense that lignin introduces a denser semicrystalline morphology to the polymers; thus, the amorphous PE zones between the crystals are further constrained by the crystals. Such a factor is capable of elevating the glass transition temperature.

#### 3.1.4. Mechanical and Morphological Characterization

**Figure 12:**
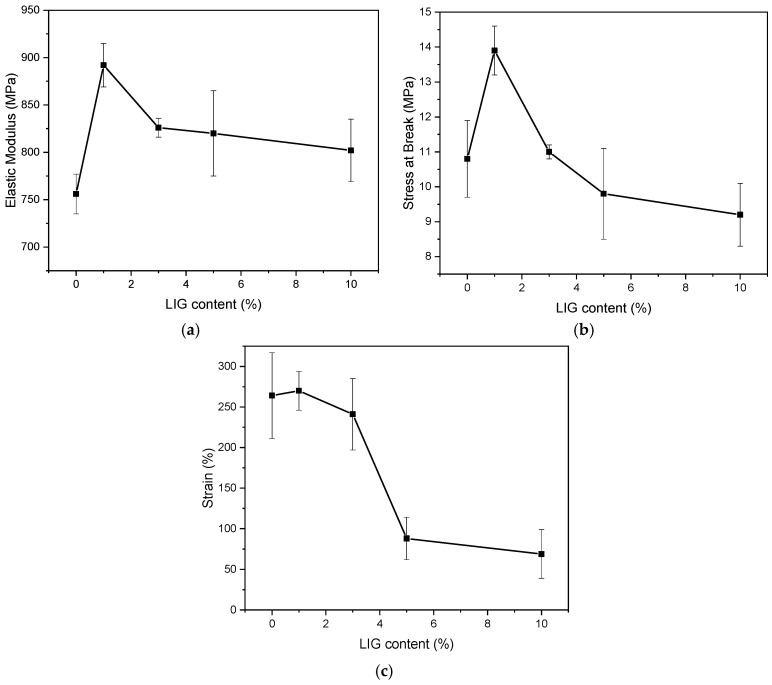
Effect of LIG content on (**a**) Young’s modulus, (**b**) stress at break, and (**c**) strain at break of the rLDPE/rHDPE composites. Lines were drawn to guide the eye.

The results from the tensile test on specimens prepared according to the ASTM standards provided insights into their mechanical behavior (Figure 12). The data shows that the additive is modifying the Young’s modulus, stress at break, and strain at break, while none of these parameters show a monotonous trend by increasing the LIG content (Table 4).

The Young’s modulus initially increases with the inclusion of LIG, reaching its peak at 1% wt. This increase implies that, at low concentrations, lignin is effectively acting as a reinforcing agent, enhancing stiffness [53]. This behavior suggests a relatively good dispersion of the additive particles and satisfactory interfacial adhesion between the polymer matrix and the LIG phase, aided by the presence of PE-g-MA compatibilizer. However, as LIG content increases beyond 1% wt, the modulus starts to slightly decline, possibly due to the onset of particle agglomeration. Such agglomerates can act as stress concentrators, hindering the reinforcing effect and reducing the efficiency of load transfer through the composite. This is further corroborated by the trend observed in tensile strength: while strength is relatively boosted at lower LIG content, it progressively decreases at higher concentrations. This decline implies that the adhesion between lignin and the matrix is insufficient to sustain mechanical loads at elevated additive contents [54,55]. Previous studies on lignin-filled polymer matrices have reported that the addition of lignin generally leads to a reduction in ductility and produces a moderate effect on stiffness due to limited interfacial compatibility [17,30].

Strain at break shows a similar pattern. The composites exhibit adequate ductility up to 1% wt LIG, indicating that the polymer chains retain sufficient mobility to elongate under tensile stress. However, by increasing the LIG concentration, even at 3% wt, the strain dramatically drops. This behavior suggests that excessive LIG loading restricts chain mobility and loss of ductility, likely due to phase separation and void formation, as seen in stereoscopic images (Figure S2).

To conclude, while lignin shows a reinforcing effect at lower loadings by enhancing stiffness, excessive addition leads to poor dispersion, weak interfacial adhesion, and ultimately the embrittlement of the composite. The role of the PE-g-MA compatibilizer is critical, yet it cannot overcome the negative effects of lignin overload. Careful optimization of lignin loading is thus essential to balance the mechanical parameters, in order to further ensure good printability for 3D-printing applications.

**Figure 13:**
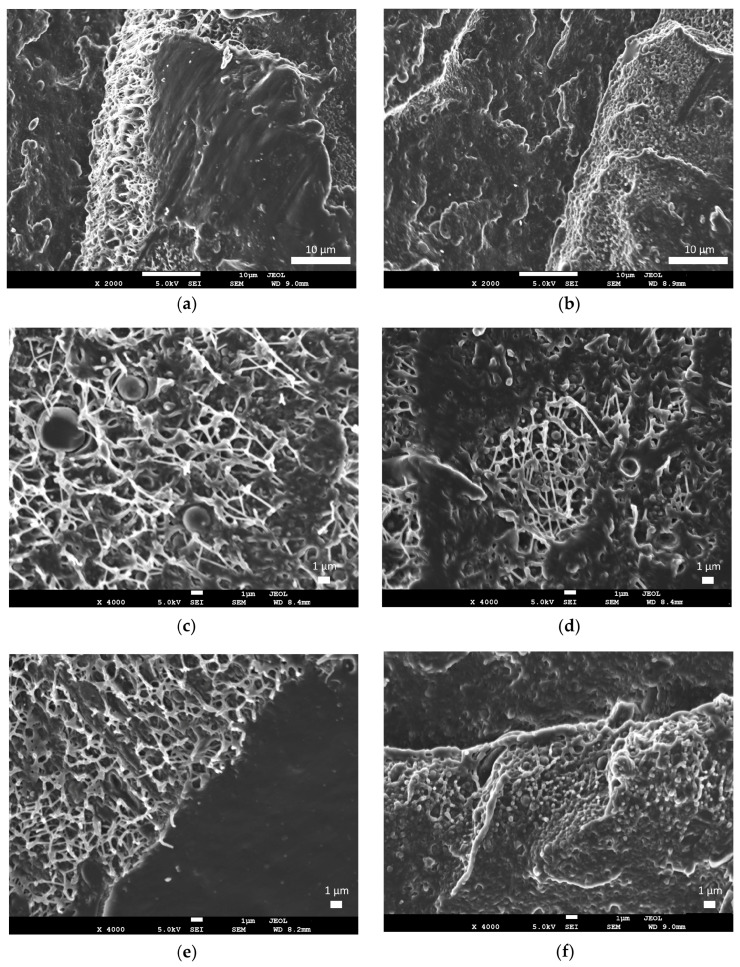
SEM micrographs of cryo-fractured surfaces of rLDPE/rHDPE-based composites: (**a**) neat rLDPE/rHDPE, (**b**) rLDPE/rHDPE/PE-g-MA, (**c**) rLDPE/rHDPE/PE-g-MA/LIG1%, (**d**) rLDPE/rHDPE/PE-g-MA/LIG3%, (**e**) rLDPE/rHDPE/PE-g-MA/LIG5%, and (**f**) rLDPE/rHDPE/PE-g-MA/LIG10%.

Scanning electron microscopy (SEM) was employed to observe the cross-section morphology and interfacial characteristics of cryo-fractured samples (Figure 13). The neat rHDPE/rLDPE blend (Figure 13a) exhibits a distinct two-phase separation with sharp interfaces, revealing weak compatibility and a heterogeneous nature. After the addition of PE-g-MA (Figure 13b), the morphology remains biphasic but notably more refined, with dispersed domains remaining attached in the matrix [56]. This suggests improved compatibility between the two polymers.

The incorporation of lignin (Figure 13c–e) induces extensive micro-scale fibrillation across the fracture plane. The presence of these fibrils, even after cryo-fracture in liquid nitrogen, indirectly suggests enhanced interfacial adhesion facilitated by the PE-g-MA compatibilizer, which forces the matrix to undergo localized plastic deformation despite the cryogenic conditions. This correlates with the optimized mechanical performance observed at 1% LIG loading. However, as the lignin concentration increases to 10% (Figure 13f), the surface exhibits a more granular appearance, closer to the island-on-sea morphology of the samples without LIG. The high density of filler particles could create stress concentration that facilitates rapid crack coalescence between adjacent particles, effectively reconciling the observed micro-scale ductile tearing with the significant reduction in elongation at break [57]. Such microstructural flaws compromise homogeneity and could account for the low mechanical integrity observed, rendering these higher-load composites unsuitable for further processing into filaments for 3D-printing.

### 3.2. Mechanical Characterization of 3D-Printed Samples

Based on the comprehensive characterization of the developed composites, the formulation containing 1% wt kraft lignin was identified as the optimal balance point for filament production. This composition exhibited improved mechanical performance, adequate thermal stability, and uniform morphology compared to other lignin loadings. To validate its performance under additive manufacturing conditions, larger batches (50 g) of both the compatibilized reference blend (rLDPE/rHDPE/PE-g-MA) and the optimized formulation (rLDPE/rHDPE/PE-g-MA/LIG1%) were prepared using the same melt mixing protocol to ensure reproducibility and consistent filler dispersion. These batches were subsequently extruded into the filament, from which tensile specimens were 3D-printed following the parameters described in the Section 2.

The mechanical properties of the printed samples were then evaluated and directly compared to the corresponding compression-molded specimens (Table 5). Overall, the printed materials followed the same trend observed previously: the incorporation of 1% wt lignin resulted in a noticeable enhancement in Young’s modulus and tensile strength relative to the neat compatibilized blend, while the strain remained unaffected. This indicates that the compatibilized system containing lignin maintains an advantageous balance between stiffness and ductility even under the more demanding conditions of filament fabrication [58,59].

As expected, the absolute mechanical values of the 3D-printed samples were slightly lower than those of their compression-molded counterparts. Such reductions are commonly associated with inherent limitations of fused filament fabrication, including the presence of micro-voids, restricted interlayer diffusion, and anisotropic layer orientation, all of which can diminish the effective load-bearing capacity of printed parts (Figure S3) [24,60]. These factors can also contribute to increased variability in the measured mechanical properties, as reflected in the relatively high standard deviation values observed [61]. These findings are consistent with previous studies on 3D-printed polyolefins, where process-related defects and limited interlayer adhesion influence the final mechanical performance [20]. Despite these constraints, the performance of the optimized formulation remained robust, demonstrating reliable printability and mechanical stability. Overall, the data suggest that the rLDPE/rHDPE/PE-g-MA/LIG1% system is a promising candidate for sustainable 3D-printing applications, combining improved mechanical performance with the valorization of both post-consumer polyolefins and bio-derived lignin.

## 4. Conclusions

In this work, sustainable composite materials based on an 80/20 rLDPE/rHDPE blend compatibilized with PE-g-MA and reinforced with kraft lignin were successfully prepared and characterized, displaying their suitability as 3D-printing filament feedstock and promoting circular economy through polyethylene waste upcycling. Structural and thermal analyses revealed that lignin incorporation has a limited effect on the crystalline structure and melting behavior of the blend, while 1% wt lignin ratio exerts a moderate nucleating impact. TGA and Py–GC/MS demonstrated that the presence of lignin maintains the overall thermal stability of the materials but also promotes char formation and redirects degradation toward a more condensed pathway, suggesting a potential for improved flame-retardant properties. The DRS analysis spotted a systematic rise in the dielectric T_g_ with increasing lignin ratio, reflecting hindered segmental mobility and denser semicrystalline morphology, which is usually assigned to efficient polymer-filler interactions. Tensile testing spotted rLDPE/rHDPE/PE-g-MA/LIG 1 wt% as the optimal sample, as it enhances Young’s modulus and tensile strength without compromising elongation at break. At a higher lignin ratio, filler agglomeration and poor interfacial adhesion lead to reduced mechanical integrity and embrittlement, as further confirmed by the SEM observations. This formulation was further extruded into a stable 1.75 mm filament and successfully FFF-printed into ASTM D638 specimens, retaining substantial reinforcement (720 MPa modulus). Τhe developed composites show potential for applications in sustainable 3D-printing, particularly when it comes to low-load products such as packaging accessories, storage components, and simple consumer goods. Considering that the raw materials originate from supermarket waste, their new applications can contribute to circular economy approaches by transforming polyolefin waste into new functional materials.

## Figures and Tables

**Table 1:** Amounts (in grams) of all components for rLDPE/rHDPE 80/20 blends reinforced with 5% wt compatibilizer and different portions of LIG.

Abbreviation	Composition (g)			
	rLDPE	rHDPE	PE-g-MA	LIG
**rLDPE/rHDPE**	8.0	2.0	-	-
**rLDPE/rHDPE/PE-g-MA**	7.6	1.9	0.5	-
**rLDPE/rHDPE/PE-g-MA/LIG1%**	7.5	1.9	0.5	0.1
**rLDPE/rHDPE/PE-g-MA/LIG3%**	7.4	1.8	0.5	0.3
**rLDPE/rHDPE/PE-g-MA/LIG5%**	7.2	1.8	0.5	0.5
**rLDPE/rHDPE/PE-g-MA/LIG10%**	6.8	1.7	0.5	1.0

**Table 2:** DSC results.

Sample	Tm (°C)	ΔHm (J/g)	Tc (°C)
**rLDPE**	123	38	110
**rHDPE**	130	104	115
**rLDPE/rHDPE**	126	43	114
**rLDPE/rHDPE/PE-g-MA**	126	66	114
**rLDPE/rHDPE/PE-g-MA/LIG1%**	126	39	114
**rLDPE/rHDPE/PE-g-MA/LIG3%**	126	44	114
**LDPE/rHDPE/PE-g-MA/LIG5%**	126	48	113

**Table 3:** Fractional composition profile of the TIC intensity foe rLDPE/rHDPE and LIG-filled composites, expressed as fractional contributions of three Rt regions, fearly (R_t_ = 0–5 min), fmid (R_t_ = 5–19 min), and flate (Rt = 19–30 min), calculated relative to the total TIC signal (R_t_ = 0–30 min).

Sample	f_early_	f_mid_	f_late_
**rLDPE/rHDPE**	0.17	0.29	0.53
**rLDPE/rHDPE/LIG1%**	0.14	0.28	0.49
**rLDPE/rHDPE/LIG10%**	0.2	0.24	0.42

**Table 4:** Mechanical parameter values—Young’s modulus, stress at break, and strain of all materials prepared—accompanied by the standard deviation.

Sample	Young’s Modulus (MPa)	Stress at Break (MPa)	Strain (%)
**rLDPE/rHDPE**	742 ± 40	9.8 ± 1.3	248 ± 20
**rLDPE/rHDPE/PE-g-MA**	756 ± 21	10.8 ± 1.1	264 ± 53
**rLDPE/rHDPE/PE-g-MA/LIG1%**	892 ± 23	13.9 ± 0.7	270 ± 24
**rLDPE/rHDPE/PE-g-MA/LIG3%**	826 ± 10	11.0 ± 0.2	241 ± 44
**rLDPE/rHDPE/PE-g-MA/LIG5%**	820 ± 45	9.9 ± 0.4	88 ± 26
**rLDPE/rHDPE/PE-g-MA/LIG10%**	802 ± 33	9.2 ± 0.9	69 ± 30

**Table 5:** Mechanical parameter values: Young’s modulus, stress at break, and strain of 3D-printed samples.

Sample	Young’s Modulus (MPa)	Stress at Break (MPa)	Strain (%)
**rLDPE/rHDPE**	688 ± 113	9.4 ± 2.0	300 ± 40
**rLDPE/rHDPE/PE-g-MA/LIG1%**	720 ± 66	12.4 ± 1.3	289 ± 14

## Data Availability

The original contributions presented in this study are included in the article. Further inquiries can be directed to the corresponding authors.

## Supplementary Materials

The following supporting information can be downloaded at: https://www.mdpi.com/article/10.3390/polym18091028/s1, Figure S1: (a) C1s photoelectron peak of LHDPE-PEgMA-1LIG, (b) C1s photoelectron peak of LHDPE-PEgMA-5LIG and (c) C1s photoelectron peak of LHDPE-PEgMA-10LIG; Figure S2: (a) O1s photoelectron peak of LHDPE-PEgMA-1LIG, (b) O1s photoelectron peak of LHDPE-PEgMA-5LIG and (c) O1s photoelectron peak of LHDPE-PEgMA-10LIG; Figure S3: (a) fracture region of the tensile specimen after elongation during tensile testing; (b) side surface of the as-printed neat blend specimen; (c) side surface of the as-printed specimen containing 1 wt% lignin. The morphology of the 3D-printed specimens was evaluated using optical stereomicroscopy. Images were captured using a digital camera and Gryphax software.
