# Supplementary material for: Development of 3D-Printing Filament from Recycled Low-Density Polyethylene (rLDPE) and High-Density Polyethylene (rHDPE) Composites Reinforced with Lignin Additive
Source: Polymers (Basel). 2026 Apr 24;18(9):1028. doi: 10.3390/polym18091028 (PMC13164592; doi:10.3390/polym18091028)
Supplement: Supplementary file 1 [file polymers-18-01028-s001.zip › polymers-4266742-supplementary.pdf]

Supplementary Information

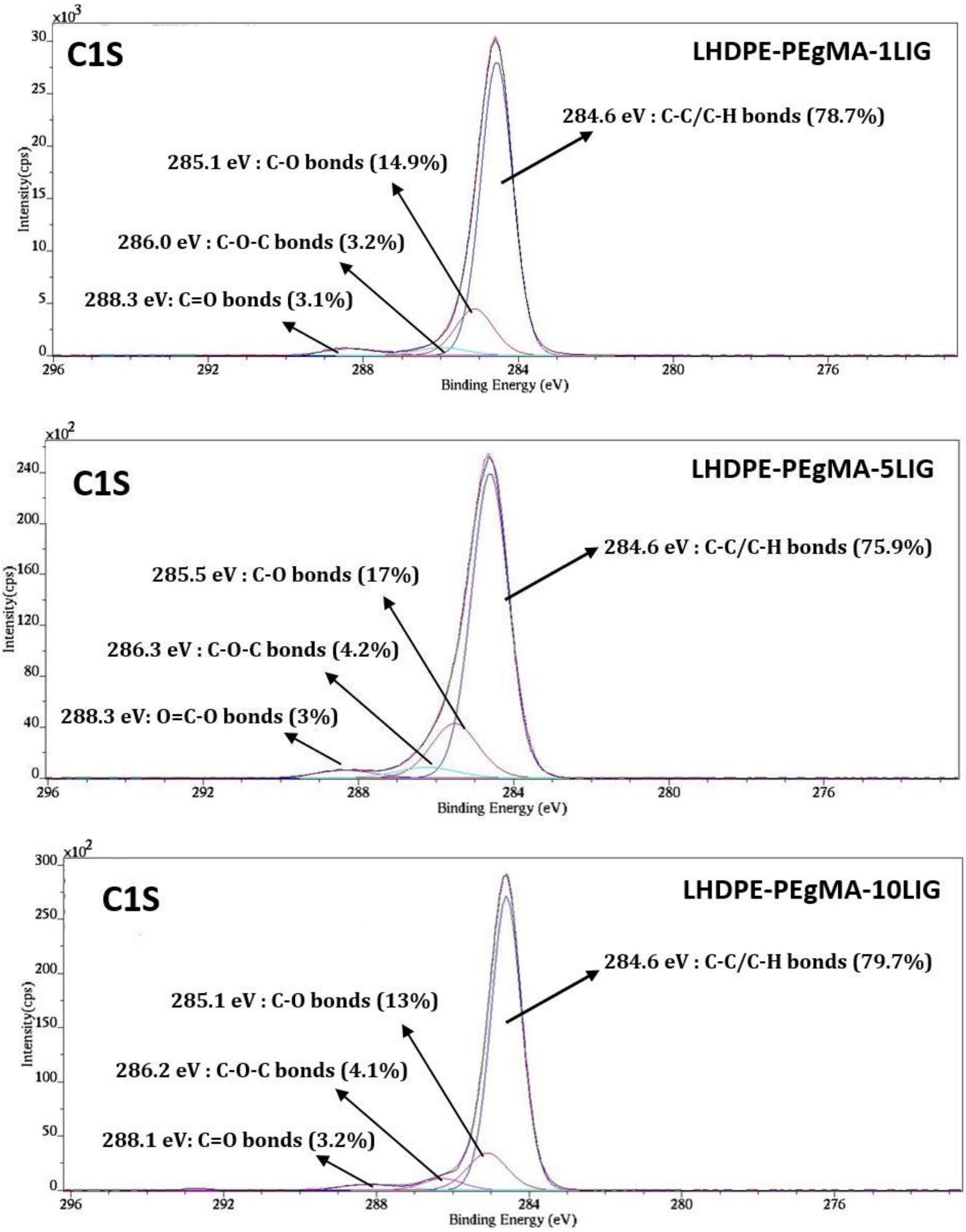

Figure S1: a) C1s photoelectron peak of LHDPE-PEgMA-1LIG, b) C1s photoelectron peak of LHDPE-PEgMA-5LIG and c) C1s 22 photoelectron peak of LHDPE-PEgMA-10LIG

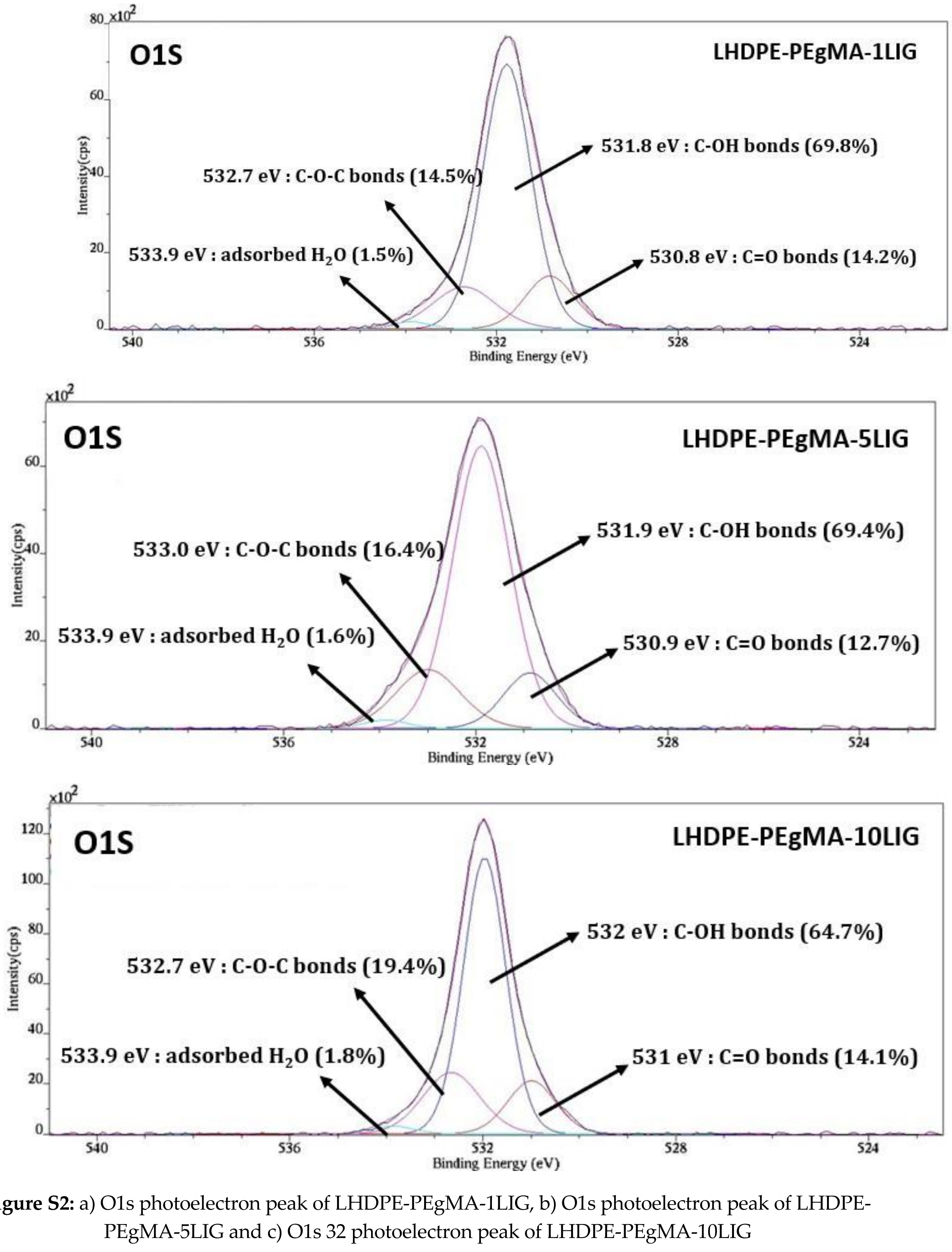

Figure S2: a) O1s photoelectron peak of LHDPE-PEgMA-1LIG, b) O1s photoelectron peak of LHDPE-PEgMA-5LIG and c) O1s 32 photoelectron peak of LHDPE-PEgMA-10LIG

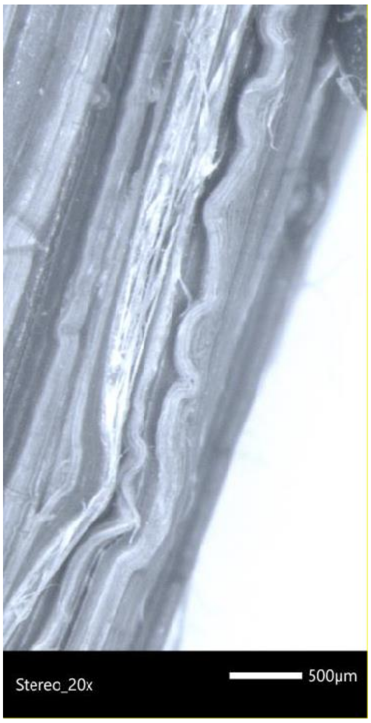

(a)

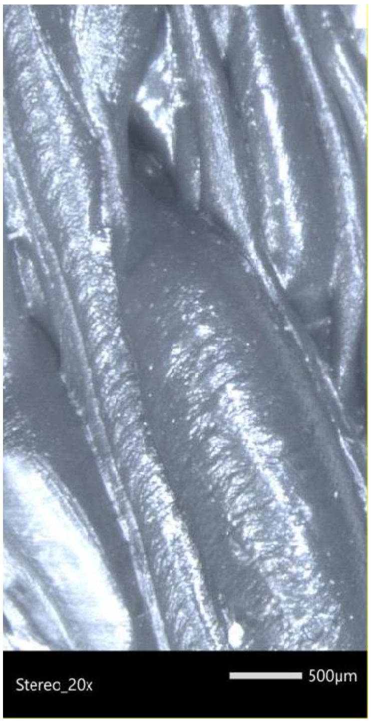

(b)

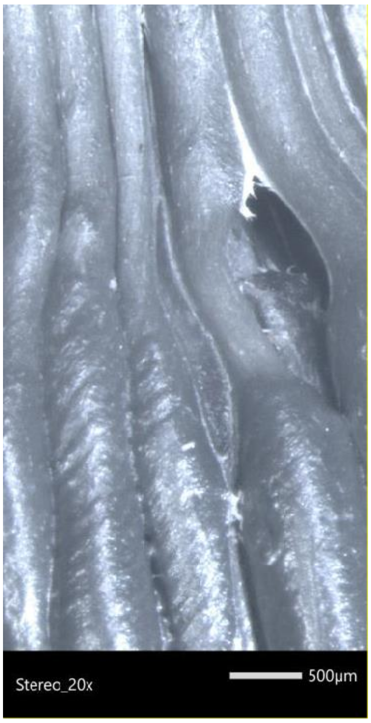

(**c**)

Figure S3: a) fracture region of the tensile specimen after elongation during tensile testing; b) side surface of the as-printed neat blend specimen; c) side surface of the as-printed specimen containing 1 wt.% lignin. The morphology of the 3D-printed specimens was evaluated using optical stereomicroscopy. Images were taken using a Jenoptik (Jena, Germany) ProgRes GRYPHAX Altair camera attached to a ZEISS (Oberkochen, Germany) SteREO Discovery V20 microscope and Gryphax image capturing software was used.
